# Effective treatment of a giant condyloma with imiquimod and HPV vaccination

**DOI:** 10.1016/j.gore.2025.101976

**Published:** 2025-10-17

**Authors:** Julia Fromme, Roland Hake, Elmar Armin Joura, Monika Hampl

**Affiliations:** a Department of Obstetrics and Gynecology, St. Elisabeth Hospital, Werthmannstrasse 1, 50935 Cologne, Germany; b Department of Pathology, St. Elisabeth Hospital, Werthmannstrasse 1, 50935 Cologne, Germany; c Department of Gynecology and Obstetrics, Comprehensive Cancer Center, Medical University of Vienna, Währinger Gürtel 18-20, 1090 Vienna, Austria; d Department of Gynecology and Obstetrics, University Hospital, Heinrich Heine University Moorenstr. 5, 40225 Düsseldorf, Germany

**Keywords:** Giant condyloma acuminatum, Imiquimod, 9vHPV vaccination

## Abstract

**Background:**

10.13039/100005130Giant condyloma acuminata (10.13039/100005839GCA) is a sexually transmitted disease caused by human papillomavirus (HPV), mostly types 6 and 11. Only a few cases have been reported in children and young patients. Treating these large tumors in adolescent patients is challenging, due to the disfiguring effects of surgery and the high risk of recurrence. Alternative nonsurgical treatments are available such as imiquimod. Imiquimod is a topically active immunomodulatory agent that is formulated as a 5% cream for application by the patient. In immunocompetent patients, imiquimod stimulates the production of interferon-alpha and various other cytokines, and has indirect antiviral activity.

**Case presentation:**

We present a case of successful treatment of an extensive GCA encompassing the entire vulva in a 14-year-old adolescent patient with a history of sexual abuse with imiquimod and three doses of nonavalent HPV vaccination (9vHPV) leading to complete remission of the GCA after 12 weeks of imiquimod application and nearly complete regression of remaining vulvar condylomas after 6 months.

**Conclusion:**

Imiquimod in combination with intramuscular 9vHPV vaccination can be an effective treatment for GCA in young patients, without the disfiguring side effects of surgery and could be considered as an alternative treatment option.

## Highlights

• Giant condyloma treated successfully with imiquimod in a 14-year-old girl.
• Nonavalent HPV vaccine used as adjunct in treatment of pediatric giant condyloma.
• Complete regression achieved without need for mutilating vulvar surgery.
• Imiquimod proved effective and well-tolerated in extensive pediatric GCA.
• Combined topical and systemic immunotherapy may offer new treatment pathway.

## 1 Introduction

Giant condyloma accuminata (GCA) is a rare tumor entity and a locally destructive variant of genital warts, characterized by large, verrucous, or cauliflower-like tumorous lesions that primarily affect the anogenital regions. Histologically, these lesions demonstrate increased mitotic activity, papillomatosis, acanthosis, and a tendency to infiltrate adjacent tissues (Spinu et al., 2014). The tumor may present as a classic condyloma but may also harbor an underlying squamous cell carcinoma (Trombetta and Place, 2001). The available literature is scarce and is largely restricted to case reports and small case series. Data suggest GCA emerges as a sexually transmitted disease following human papillomavirus (HPV) infection. Other routes of transmission are rare. The low-risk HPV types 6 and 11 are most commonly responsible for GCA, although other subtypes have also been reported.

The treatment of these extensive tumors is challenging, particulary in children and young adults. It may be painful and disfiguring, and the risk of recurrence is high (Nieves-Condoy et al., 2021). Multiple treatments for adults have been reported in the literature, such as topical or systemic chemotherapy, cryotherapy, radiation therapy, application of intratumoral vaccines, or imiquimod (5 % cream) has shown to be effective treatment (Nieves-Condoy et al., 2021). The most common treatments are extensive surgical resection and laser treatment (Spinu et al., 2014). For pediatric patients, treatment with imiquimod, trichloroacetic acid (TCA), and podophyllotoxin has been documented (Dinleyici et al., 2015, Hum et al., 2018, Suarez-Ibariola et al., 2016, Gianchristoforo et al., 2020).

## 2 Case presentation

We present the case of a 14-year-old female patient who underwent successful treatment for GCA with imiquimod and a secondary nonavalent HPV (9vHPV)-vaccination (Gardasil 9, Merck, USA).

The patient first presented to our colposcopy and vulvar clinic, St. Elisabeth Hospital in Cologne in 2024. She had left Guinea as a refugee 14 months ago without her family. During her seven-month journey, which included a two-month detention in Libya, she was imprisoned and reported experiencing abuse. Upon arrival in Germany, she was placed in the care of the youth welfare office.

The admitting physician performed a gynecological examination due to complaints of genital pain and itching, and a tumor mass with a three-months history. The examination confirmed a large exophytic tumor covering the entire vulva. Sexually transmitted infections (STIs), including HIV, were ruled out during the gynecological examination, and a biopsy of the tumor was taken. Histology reported a condylomata acuminata. Due to the size of the lesion, the patient was referred to our vulvar clinic for further diagnosis and treatment.

**Fig. 1:**
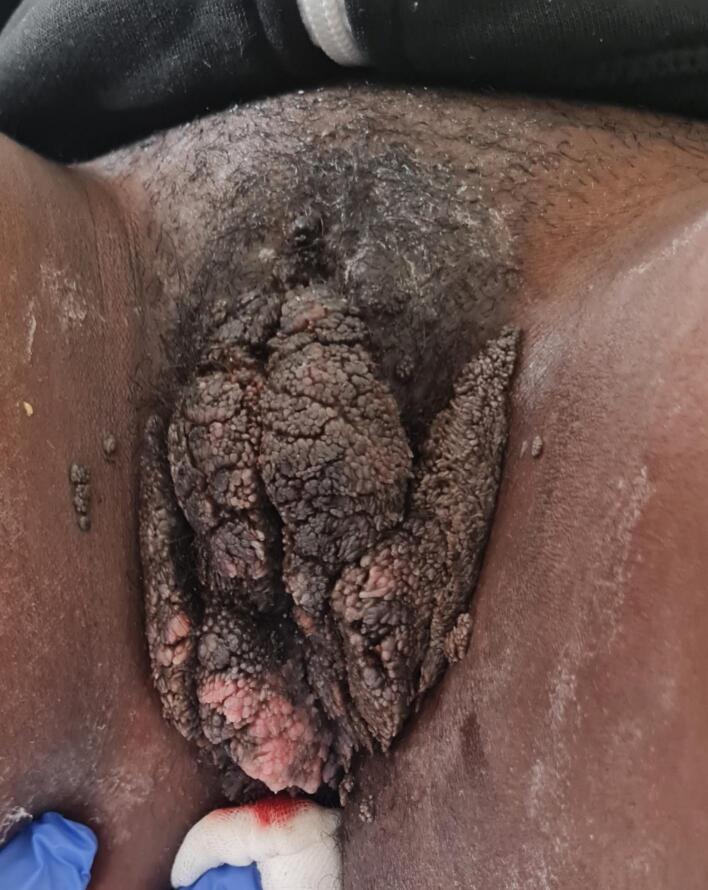
Exophytic vulvar tumor (approx. 12 x 9 cm) in 14-year-old female patient at first presentation.

During gynecological examination at initial presentation, inspection of the vulva revealed an extensive condylomatous mass measuring 12 x 9 cm, extending from the pubic area to the perineum and from the inguinal folds to the thighs, completely covering the labia majora bilaterally (Fig. 1). A speculum examination was not possible due to the patient’s pain and discomfort. The introitus was filled with condylomatous tumor masses.

An STD and HPV screening panel had already been performed (Chlamydia, Gonorrhea, Syphilis, HIV negative; Ureaplasma urealyticum, Mycoplasma hominis and Ureaplasma parvum positive; HPV 18 and 6/11 DNA was present in the tumor). To obtain a second opinion on the histological examination, a re-biopsy of the vulvar tumor was performed after receiving the patient’s consent.

Biopsy sections revealed a squamous epithelium with papillary architecture, abundant hyperkeratosis, and marked koilocytosis, typical of condyloma. There was no overexpression of p16, Ki67 proliferation was 20 %. The pHH3 staining showed only a slightly increased mitotic activity, with no evidence of malignant cell degeneration. Taking the clinical picture into account, the findings are compatible with a GCA.

## 3 Treatment

Due to the size and extent of the tumor, a surgical intervention would have required extensive tissue resection in this 14-year-old patient. Preservation of the vulva would not have been feasible and it remained unclear whether the tumor extended into the vagina. Surgery would have been associated with loss of vulvar anatomy, substantial pain, risk of impaired wound healing and potentially recurrent disease. Her young age, short disease duration, an overall good health made her a suitable candidate for combining local and systemic therapy.

Taking into account published cases in the literature on the successful treatment of GCA in adolescents using imiquimod, we initiated an off-label treatment for 12 weeks. The imiquimod 5 % cream was applied three times a week in the evening and removed with water in the morning. Additionally, a nonavalent HPV (9vHPV)-vaccination was initiated immediately, with subsequent doses administered at the standard two- and six-month intervals as an individualized decision.

The patient returned for another appointment three months later. She tolerated the imiquimod therapy well; no fever or flu-like symptoms occurred, and only moderate pain was reported. She was adherent to the prescribed regimen. During the therapy, the patient had manually removed locally detaching tumor masses by herself due to pronounced itching.

In addition, she had received and well tolerated two injections of the 9vHPV vaccine.

**Fig. 2:**
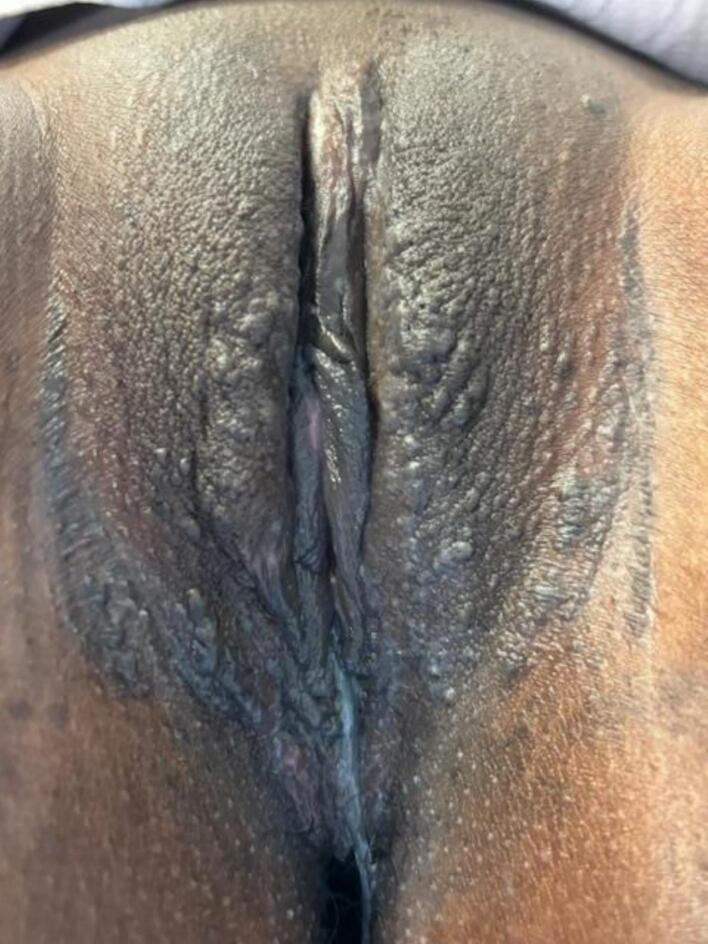
Vulva of the 14-year-old adolescent patient after 12 weeks of imiquimod application three times per week and two injections of the 9vHPV vaccine.

The examination after 12 weeks revealed that the tumor had completely regressed locally (Fig. 2); only a few small condylomas were visible on the labia majora.

We recommended continuing the imiquimod treatment for another 8–10 weeks. The HPV vaccination series should be completed with a third injection after six months.

The patient returned after another 10 weeks (six months after initial presentation) for a follow-up appointment, still undergoing imiquimod treatment. The patient only reported persistent itching of the perineum and the interlabial sulcus.

After six month of local imiquimod treatment and three doses of 9vHPV vaccination, colposcopic examination of the vulva revealed external genitalia of normal appearance, with no signs of residual tumor. A few condylomas were noted with a delicate aceto-white mucosal reaction indicative of persistent subclinical HPV infection.

After obtaining consent, colposcopic examination was performed showing a small cervix with an unremarkable transformation zone type 1, with no pathognomonic changes after application of 3 % acetic acid. A Pap smear and HPV testing revealed a low grade lesion (LSIL/ Pap III D1) and an HPV 18 infection of the cervix, which is consistent with the patient́s history and the presence of HPV-18 DNA in the vulvar tumor swab.

Given the minimal residual condylomas, we recommended discontinuing imiquimod treatment and arranged a follow-up appointment six month later. Unfortunately the patient was lost to follow-up, and no further contact through the authorities could be established.

## 4 Discussion

Treatment modalities in GCA are often surgical resection alone or in combination with topical agents (e.g., 5-fluorouracil or podophyllin), as well as systemic chemotherapy, imiquimod therapy (Dinleyici et al., 2015, Suarez-Ibarrola et al., 2016, Hum et al., 2018), or radiation therapy (Nieves-Condoy et al., 2021). The surgical approach is preferred in adults because of the potential for underlying invasive carcinoma. However, the reported recurrence rates in adults are high, reaching up to 66 % overall and approximately 50 % even following radical surgery, with a mortality rate of 20 % (Chu et al., 1994 Sep, Nieves-Condoy et al., 2021). A recent retrospective analysis of 38 cases from Zhang et al. in 2020 reported a recurrence rate of 23.7 % after a median follow up of 23 months and a disease specific mortality of 2.6 %. These data are limited to retrospective case series in adults, with no reports in children (Zhang et al., 2020).

Topical agents such as imiquimod are well-known to be highly effective in treating common genital warts. It is known that imiquimod activates immune cells that stimulates the production of interferon-alpha, tumor necrosis factor, and other cytokines that can inhibit viral replication. Imiquimod also enhances cell-mediated immunity by activating Langerhans cells and enhancing antigen presentation (Dinleyici et al, 2015). Although imiquimod is not licensed for children, there are reports of successful applications in pediatric patients with classical condyloma and GCA (Giancristoforo et al., 2020), with good tolerability and only transient, moderate side effects (Lacey et al., 2013).

However, there is little evidence concerning the effectiveness of imiquimod against GCA. Only a few case reports describe the efficacy of imiquimod alone, or in combination for treating GCA in children (Dinleyici et al., 2015, Suarez-Ibariola et al., 2016, Gianchristoforo et al., 2020). The report of Suarez-Ibarrola et al. notes the failure of imiquimod as an initial monotherapy in one case. In the other two cases, imiquimod was used as an adjuvant therapy after surgery for persistent warts. Dinleyici et al. reported complete clearance with imiquimod monotherapy in a 2-year-old child. Giancristoforo et al. used imiquimod 5 % in a 2-year-old female patient with a giant anal condyloma and achieved complete remission after five months. Several case reports in adults also report that imiquimod is a successful treatment option for this age group (Hum et al., 2018, Combaud et al., 2018). Using imiquimod over surgery may help to avoid extensive vulvectomy, or at least reduce the amount of tissue that needs to be excised, thereby avoiding loss of vulvar anatomy in children.

Based on the literature, we initiated treatment with imiquimod 5 % in combination with 9vHPV-vaccine as an individualized approach for our 14-year-old patient to avoid disfiguring surgery with unpredictable consequences for her future sexual life. Recognizing that HPV vaccination does not treat existing HPV infection but rather prevents new HPV infections and potentially autoinoculation (Reuschenbach et al., 2023), we initiated HPV vaccination in combination with imiquimod treatment in expectation of an immune response against HPV 6/11. A recent review summarized the the potential role of intramuscular and intralesional HPV vaccination in preventing and treating common, plantar, recalcitrant and anogenital warts (AGWs), however the results concerning a therapeutic effect were inconclusive (Ong et al., 2025): Several prospective studies with intramuscular HPV vaccination have been performed for the treatment of anogenital warts (AGWs), but not GCA. A small observational trial in 10 patients reported a 60 % complete response (CR) rate (Choi, 2019). Other studies used intramuscular 9vHPV vaccine in combination with other therapies (cryotherapy or imiquimod). A small matched pair study demonstrated CR in 64 % of patients in the 9vHPV combination group compared to 25 % in the standard treatment group a statistically significant result in favour of vaccination (Ciccarese et al., 2023). In contrast, a randomized trial of 503 patients with AGWs (hipvac factorial RCT) comparing the efficacy of imiquimod or podophyllotoxin creams, with or without intramuscular 4vHPV, found that vaccine addition did not result in significant differences in wart clearance (OR = 1,30 (0.89–1.91)) or recurrence (OR = 1.39 (0.73–2.63)) (Gilson et al., 2020). Whether systemic 9vHPV vaccination contributed to the successful treatment in our case, in addition to imiquimod therapy, remains an unanswered question.

A direct antiviral, antitumor, or an immunologic mechanism may have contributed to the outcome in our 14-year-old patient, similar to the effect reported by Nichols AJ et al. in case of a renal transplant recipient with squamous cell carcinoma that was treated with systematic and intratumoral HPV vaccination (Nichols et al, 2020). Another report described successful treatment of GCA with intramuscular and intralesional HPV vaccine application in a patient with pemphigus vegetans (Thomas et al., 2022). Our case adds to the limited literature, representing successful treatment of a 14-year-old patient with adjuvant 9vHPV vaccination in combination with imiquimod.

Although acute medical problems can often be effectively addressed, ensuring long-term adherence to follow-up care for adolescents with complex health needs is challenging. These patients frequently require coordinated assistance that extends beyond the purely medical domain, so psychosocial support is a crucial component. Continuity of care may be hindered by limited resources, fragmented service structures, a lack of age-appropriate psychosocial interventions, or difficulties establishing a stable therapeutic alliance. Addressing these challenges requires multidisciplinary collaboration, culturally and developmentally sensitive approaches, and flexible care models that can respond to individual psychosocial circumstances.

In conclusion, GCA remains a rare entity with variable presentations without established treatment guidelines. Thus, management of this disease with imiquimod and HPV vaccination can be an effective approach in lieu of surgery and may be tailored to each patient’s needs, particulary in young patients with potentially highly responsive immune system.

## CRediT authorship contribution statement

**Julia Fromme:** Writing – original draft. **Roland Hake:** Writing – review & editing, Investigation. **Elmar Armin Joura:** Writing – review & editing. **Monika Hampl:** Writing – review & editing, Supervision, Conceptualization.

## Informed consent

Authors obtained written and signed consent to publish the case with pictures from the legal guardian. A copy of the written consent form is available for review upon request.

## Declaration of competing interest

The authors declare that they have no known competing financial interests or personal relationships that could have appeared to influence the work reported in the paper
